# The German COVID-19 Digital Contact Tracing App: A Socioeconomic Evaluation

**DOI:** 10.3390/ijerph192114318

**Published:** 2022-11-02

**Authors:** Stephan Ellmann, Markus Maryschok, Oliver Schöffski, Martin Emmert

**Affiliations:** 1 Department of Radiology, Friedrich-Alexander-Universität Erlangen-Nürnberg, University Hospital Erlangen, Maximiliansplatz 3, 91054 Erlangen, Germany; 2 School of Business, Economics and Society, Chair for Health Management, Friedrich-Alexander-Universität Erlangen-Nürnberg, Lange Gasse 20, 90403 Nürnberg, Germany; 3 Faculty of Law, Business and Economics, Institute for Healthcare Management and Health Sciences, University of Bayreuth, Prieserstraße 2, 95444 Bayreuth, Germany

**Keywords:** COVID-19, digital contact tracing, Corona Warn App, utility analysis, cost–benefit analysis

## Abstract

The COVID-19 pandemic posed challenges to governments in terms of contact tracing. Like many other countries, Germany introduced a mobile-phone-based digital contact tracing solution (“Corona Warn App”; CWA) in June 2020. At the time of its release, however, it was hard to assess how effective such a solution would be, and a political and societal debate arose regarding its efficiency, also in light of its high costs. This study aimed to analyze the effectiveness of the CWA, considering prevented infections, hospitalizations, intensive care treatments, and deaths. In addition, its efficiency was to be assessed from a monetary point of view, and factors with a significant influence on the effectiveness and efficiency of the CWA were to be determined. Mathematical and statistical modeling was used to calculate infection cases prevented by the CWA, along with the numbers of prevented complications (hospitalizations, intensive care treatments, deaths) using publicly available CWA download numbers and incidences over time. The monetized benefits of these prevented cases were quantified and offset against the costs incurred. Sensitivity analysis was used to identify factors critically influencing these parameters. Between June 2020 and April 2022, the CWA prevented 1.41 million infections, 17,200 hospitalizations, 4600 intensive care treatments, and 7200 deaths. After offsetting costs and benefits, the CWA had a net present value of EUR 765 m in April 2022. Both the effectiveness and efficiency of the CWA are decisively and disproportionately positively influenced by the highest possible adoption rate among the population and a high rate of positive infection test results shared via the CWA.

## 1. Introduction

The COVID-19 pandemic (Coronavirus Disease 2019) has challenged governments around the world due to the pre-symptomatic transmission and short generation times of the virus [1] and has questioned traditional containment measures based on purely symptomatic surveillance. An early model study [2] proposed using mobile apps for pandemic mitigation that log and report encounters between infected individuals to prevent onward transmission. Early implementations of this so-called digital contact tracing (DCT) in Singapore and South Korea [3] had encouraged more than 40 countries to also introduce DCT apps by the end of 2020.

In Germany, as well, there were timely efforts to launch such an app (in the following for Germany: CWA—Corona Warn App), which was released on 16 June 2020. As a particular feature in an international comparison, the German CWA can be used completely anonymously and entirely refrains from localizing users. Infected CWA users merely send pseudonymized IDs to a central server, which are then downloaded by all other CWA users. The actual check for a relevant contact takes place exclusively locally on the mobile devices. Compared to other European DCT solutions, the costs for the development and operation of the CWA were enormous at over 130 million euros between June 2020 and April 2022 [4,5]. These high costs also led to criticism, especially from opposition parties, who called for the CWA to be shut down directly after its release.

Against the background of the high costs and the potential benefits, which could only be vaguely defined at the time of the app’s release, criticism voiced at that time may have been justified. In the meantime, however, sufficient data are available to objectively put the CWA to the test. There already is a substantial number of studies that investigated the privacy design of DCT apps [6,7], answered the question of which social groups use DCT from a sociological point of view [6,8,9], and also analyzed the theoretical effectiveness of such solutions [2,10,11,12,13]. Nevertheless, to our knowledge, there are currently no studies that attempt to estimate the impact of such apps on case numbers over the course of the pandemic, nor are there any studies that relate costs and benefits from a financial perspective, neither generally in an international context, nor specifically for the German CWA.

The aim of this paper is therefore to analyze the CWA in terms of health economics from the perspective of the German society as a whole and to identify factors significantly influencing its effectiveness and efficiency. The applied model calculations are based on real values as far as possible and, in the absence of real values, on justifiable plausible assumptions. The analysis is conducted in four steps:

1. First, descriptive statistics provide information on how the CWA and its use have developed among the German population.
2. Then, a utility analysis examines the effectiveness of the CWA. According to the federal government, the stated goal of the CWA is to quickly detect and interrupt chains of infection [14]. Whether it has met this goal is analyzed by modeling prevented infections, hospitalizations, intensive care treatments, and deaths.
3. In the third step, the efficiency of the CWA is examined by means of a cost–benefit analysis. A societal economic welfare criterion is determined from monetized and summed effects and quantified via net present value (NPV) and benefit–cost ratio (BCR).
4. The fourth step focuses on identifying and quantifying the factors with a crucial impact on the effectiveness and efficiency of the CWA.

The results of this study may help inform potential or already active users about the benefits of using the CWA for themselves and society and provide CWA developers with insights into critical variables affecting the efficiency and effectiveness of the CWA. Moreover, the results of this study could potentially enable policymakers, public health departments and health ministries, funders, and ultimately taxpayers to put the substantial costs of the CWA into perspective with its benefits.

## 2. Materials and Methods

### 2.1. Software and Statistics

All calculations were performed using Microsoft Excel for Mac version 16.59 (Microsoft Corporation, Redmond, DC, USA) and GraphPad Prism for Mac 8.4.3 (GraphPad Software, San Diego, CA, USA). Confidence intervals (CIs) were calculated at a 95% confidence level.

### 2.2. Actuarial Assumptions

The cut-off date for the subsequent evaluation was defined as 1 April 2022. All costs incurred and all (monetized) benefits accrued during the app’s release and the cut-off date were therefore discounted with reference to 1 April 2022. The terminal value of an amount as of 1 April 2022 is thus dependent on its amount on the date of booking, the time difference from the booking date to the cut-off date, and the assumed interest rate of 0.7% [15].

### 2.3. Calculation of the Effectiveness of the CWA

A factor with an important impact on the effectiveness of the CWA is the widest possible use by the population. If, for example, 10% of the population uses the CWA (adoption rate 10%), the probability that an infected person belongs to this group is also 10%. If this person has contact with another person who also belongs to the circle of users with a probability of 10%, the CWA would be able to uncover this potential chain of infection. The probability would be 10% × 10% = 1% for the described situation, i.e., one out of 100 potentially infectious contacts would be detected by the CWA [16]. Similarly, with an adoption rate of 50%, 25% (=50% × 50%) of the contacts would be detected by the CWA. Since both the infecting and the infected person need to use the CWA for transmission to be tracked, the effectiveness of such apps is generally assumed to be quadratically dependent on the adoption rate [17].

The cumulative download numbers of the CWA are published as a time series by the German Robert Koch Institut (RKI) [18] and can be related to the total population of Germany (83.2 million inhabitants). The proportion of active users in relation to the total number of downloads is estimated at 59.9% (95% CI: 53.9–65.9%) [19]. However, the CWA does not automatically share recorded positive test results but asks users for permission beforehand. This permission is granted by users in 60.1% of cases [20]. 

Since it is irrelevant to the person being warned whether they themselves would share their own possibly positive test result, but plays a decisive role for the person being warned, the effective adoption rate β_eff_ of the CWA on each day of the period under investigation can be expressed as follows:

(1) $$\beta_{\mathrm{eff}}=\sqrt{{\left(\frac{\mathrm{Cumulative}\;\mathrm{Downloads}}{83.2\times 10^{6}}\times 59.9\%\right)}^{2}\times 60.1\%}$$

### 2.4. Reduction of the R-Value and the Number of Cases by the Corona Warn App

An important consideration in evaluating the CWA is its potential to reduce the R-value. The R-value indicates the average number of secondary infections caused by a single case of infection and is regarded as one of the most important parameters for monitoring pandemics and epidemics. It is thus often used to evaluate the effectiveness of measures [21]. 

During the course of a pan-/epidemic, the effective reproductive number R_eff_ is of critical relevance. R_eff_ changes as the population becomes increasingly immunized, either through individual immunity after surviving infection, through vaccination, or through increasing deaths. Furthermore, R_eff_ depends on the number of individuals with whom infected individuals are in contact and thus on voluntary measures such as social distancing or government-imposed measures such as lockdowns [22,23]. 

**Figure 1:**
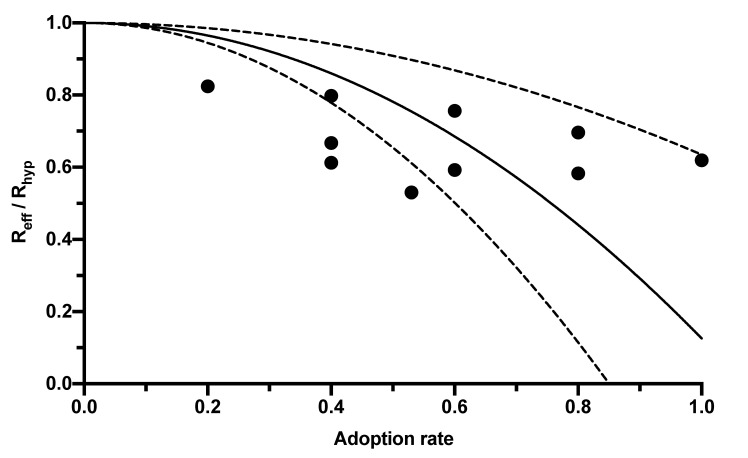
Relationship between the reduction in the R-value and the adoption rate of digital contact tracing apps. The black dots mark the results of the individual studies reported in Table 1. The solid line corresponds to the regression curve (y = 1 − β^2^ × 0.8741). The dashed lines mark the 95% CI.

The CWA, as an additional measure, also has the potential to further reduce R_eff_. This potential depends on its adoption rate among the population. However, the range of adoption rates published in the literature and the associated R_eff_ reductions is broad, so a regression analysis was created using the correlations published in four studies (Table 1) to express the reduction in R-value by the CWA as a function of its adoption rate (Figure 1).

The dependency of the R-value reduction on the adoption rate can be expressed by the following equation:

(2) $$R_{\mathrm{eff}}=R_{\mathrm{hyp}}\times \left(1-{\beta_{\mathrm{eff}}}^{2}\times f\right),\;\mathrm{or}\;\frac{R_{\mathrm{eff}}}{R_{\mathrm{hyp}}}=\left(1-{\beta_{\mathrm{eff}}}^{2}\times f\right),\;\mathrm{or}\;R_{\mathrm{hyp}}=\frac{R_{\mathrm{eff}}}{1-{\beta_{\mathrm{eff}}}^{2}\times f}$$

In this equation, R_eff_ represents the R-value given the population using the app (and thus corresponds to the R-value published by the RKI, which is lower than the hypothetical R-value R_hyp_ without app usage). The adoption rate is symbolized by β_eff_, and f is a constant combining further influencing factors, such as delays between CWA notification and testing, between testing and its result’s entry into the CWA, compliance with measures such as self-quarantine, and the proportion of pre- or asymptomatic infections. The value of this constant calculated in the regression analysis was 0.8741 (95% CI 0.3651–1.383; Figure 1).

**Figure 2:**
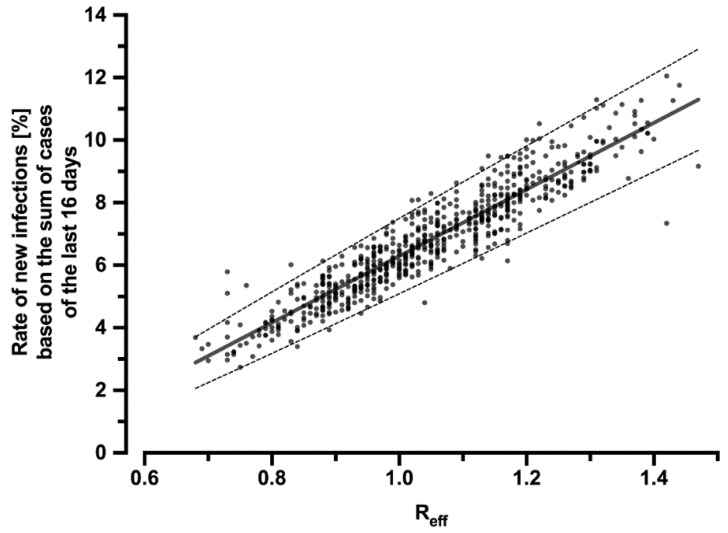
Correlation of the effective reproduction number R_eff_ with the percentage rate of new infections related to the sum of cases over the last 16 days. The regression line can be described by the equation y = 0.1064x − 0.04353 (R^2^ = 0.87) and is symbolized by the solid line. The dashed lines indicate the 95% CI (slope: 0.1034–0.1095; y-intercept: −0.04669–−0.04038). The dots represent the individual values for each day during the period under consideration.

To infer how the reduction of R_eff_ by the CWA affects the number of cases, the correlation of R_eff_ with the percentage rate of new infections in relation to the sum of new infections of the past 16 days was calculated (Figure 2):

(3) y=0.1064 x−0.04353

Thus, using the sum of new infections over the past 16 days and the hypothetical R-value R_hyp_ (which would have occurred without the CWA), the number of new infections that would have resulted had the CWA not been used can be calculated. The difference between this hypothetical number of cases and the real, published number of cases yields the reduction in infection cases due to the CWA.

### 2.5. Cost Calculation

#### 2.5.1. Development and Operation of the CWA

The basis for calculating the costs incurred for the development and operation of the CWA was the German government’s response to a minor inquiry [5].

According to this, the CWA cost EUR 52.8 million in 2020. Of this, EUR 20 million was for pure development, which was booked to the app’s release date (16 June 2020). The remaining EUR 32.8 million was booked on the first of the months of 2020, corresponding to EUR 5.47 million per first of the month.

Further development and operations were reported at EUR 63.5 million in the fiscal year 2021, with EUR 5.29 million booked to the first of each month in 2021. The monthly operating costs from 2022 onward were stated at EUR 2.36 million and were also booked on the first day of the month.

Press costs up to the end of 2021 were estimated at EUR 13.7 million, corresponding to a monthly amount of EUR 721,053, booked on the last day of each month and continued for 2022, assuming constancy. The digital advertising costs of EUR 71,000 were handled in the same way as a monthly amount of EUR 3737 at the penultimate of each month.

#### 2.5.2. Costs of Testing

Users who receive a red warning from the CWA are entitled to a test for infection. This option is taken up by 87% of users [24]. While positive tests detect infections and thus help reduce further transmissions, negative test results simply provide feedback to the person tested that the red alert was a contact that, in their case, did not result in infection. However, since these tests, which can be classified as negative ex post, were performed because of the CWA warning, the CWA in these cases incurred costs that were not directly offset by added value. These costs were determined as follows:

The numbers of tests and their positivity rates are available as a time series [18,25]. The number of red alerts received is available as a time series from 3 June 2021 [18]. For the period until 3 May 2021, the number of red alerts received was therefore extrapolated as it correlates linearly with the total number of cases (y = 0.189 x; R^2^ = 0.83), which is consistently published by the RKI. However, for privacy reasons, the published number of red alerts received corresponds only to those users who voluntarily activated the app’s data donation option. This percentage is estimated at 58.6% [19]. 

Test costs were calculated using ratios of rapid tests to PCRs [18,25] and their unit costs [26,27] as a weighted average.

#### 2.5.3. Costs Due to Continued Pension Payments

The reduction in the number of cases caused by the CWA also results in a reduction in COVID-19-related deaths. Prevented deaths of retirees lead to costs in the form of continued pension payments. 

The case fatality rate (CFR) was calculated from published deaths [28] in relation to infection cases [29] and multiplied by the number of cases reduced by the CWA to obtain the number of deaths prevented by the CWA. Retirees accounted for 89.5% of these reduced deaths [30]. Their average monthly pension was assumed to be EUR 1012 [31,32], and the total cost was calculated as the product of the pensioners’ lifetime saved during the period under consideration and their average pensions. 

However, because retirees are also subject to a non-negligible risk of dying independently of COVID-19 infections in the period between each day of the analysis and the cut-off date of 1 April 2022, this risk was also appropriately accounted for—because in these cases, pension continuation will only be provided until the date of death occurring independently of COVID-19 infection. This probability can be calculated for a person older than 65 years from the death rates by age and sex and their percentage distribution at 4.96% per year [33,34] and adjusted to the probability valid on each day until 1 April 2022. With these assumptions, it was possible to calculate the pension continuation costs of the deaths prevented on each day of the evaluation period.

The costs for the CWA, for testing and for pension payments, which were calculated on a daily basis, were discounted to 1 April 2022.

### 2.6. Benefit Calculation

#### 2.6.1. Benefits from Reduced Loss of Earnings

COVID-19 infection causes—in terms of lost earnings due to incapacity for work (IfW), which affects gross domestic product (GDP). Consequently, the prevention of infection by the CWA contributes to a reduced burden on GDP. 

The GDP per capita in Germany was set at EUR 36,600 per year [35]. At a minimum, infection leads to isolation with IfW, and in more severe cases, to hospitalization, intensive care treatment, or death. While the latter three cases cause further costs, which will be calculated in more detail below, all cases of infection among employed persons result in a reduction in GDP.

Based on first-wave data, the median duration of hospitalization was 9 days (interquartile range 4–17 days), and the median duration of intensive care was also 9 days (interquartile range 4–18 days) [36]. After discharge, however, these patients are not able to resume work immediately—the median duration of illness for symptomatic patients is reported to be 27.5 days [37,38]. However, 40.5% of infections are asymptomatic [39], so for these patients, the duration of illness, and thus IfW duration, can essentially be equated with the prescribed duration of isolation, which was 14 days for much of the period under consideration [40,41]. Thus, the duration of IfW was assumed to be a weighted average of 22 days. In line with these considerations, international studies also estimate the time to recovery to be 21–22 days on average [42,43,44].

Since there is an effect on GDP only in the case of employed persons, the proportion of cases in this age group was calculated on the assumption of a retirement age of 65 and an occupational entry age of 20. This proportion varied between 58.0% and 74.8% over the course of the pandemic [41]. 

With these assumptions, it was possible to calculate the monetary benefits from prevented IfW-related negative impacts on GDP.

#### 2.6.2. Benefits from Reduced Hospitalizations and Intensive Care Treatments

According to insurers’ data, patients in normal wards incurred average costs of EUR 6600 per case and intensive care patients EUR 26,000 [45].

To calculate the costs saved, the percentage of hospitalizations [46] and admissions to intensive care units [47] were multiplied by the cases of infection prevented by the CWA and the corresponding case costs.

#### 2.6.3. Benefits from Reduced Rehabilitation Measures

Non-retired patients account for 85.2% of COVID-related rehabilitation treatments, with a single treatment lasting an average of 27 days [48]. There is considerable uncertainty regarding the number of patients requiring rehabilitation after COVID-19 infection, but it is assumed below that every patient requiring intensive care and 50% of regularly hospitalized patients will require a rehabilitation treatment after discharge.

The daily rate was set as an average of the rates for cardiological and geriatric rehabilitation at EUR 211 [48]. With these assumptions, it was possible to estimate the number of rehabilitation measures prevented by the CWA and the associated benefits through saved costs (including GDP effects in the case of working people).

#### 2.6.4. Benefits from Reduced Deaths

A reduction in the number of cases due to the CWA also implies a reduction in the number of deaths. If the deaths prevented are part of the working population, preventing their deaths translates into their ability to continue contributing to GDP after recovery. Assuming that people aged 20–65 contribute to GDP as working people, only the reduction in deaths within this age group is also GDP-relevant. COVID-19-related deaths in this age group can be assumed to account for 10.3% of the total number of deaths [33]. 

Thus, the monetized benefit of reduced GDP-relevant deaths is equal to the product of GDP (adjusted to the period up to the cut-off date), the number of reduced deaths, and the factor 0.103.

The monetized benefits from prevented IfW, hospitalizations, intensive care treatments, rehabilitations, and deaths calculated on each day were discounted relative to 1 April 2022.

### 2.7. Net Present Value and Benefit–Cost Ratio

Net present value (NPV) and benefit–cost ratio (BCR) were applied as indicators of total costs and monetary benefits.

### 2.8. Sensitivity Analysis

With the above assumptions, a basic model was developed to calculate the costs and benefits of the CWA. Subsequently, this basic model was modified in a sensitivity analysis by subjecting the most important parameters of the model to random fluctuations (Table S1). In this way, 1500 additional models were calculated and analyzed in their entirety to better reflect the possible uncertainties of original estimators. 

In detail, lower and upper limits were set for the relevant parameters, and random fluctuations were allowed within these limits. For pure estimators (such as the cost of hospital or intensive care treatment), upper and lower limits were defined as ±10% variation around the base model estimator. For estimators such as the R-value or the number of cases, for which 95% confidence intervals were already available, these confidence intervals were used as upper and lower limits.

However, these upper and lower limits were not fixed rigidly but were defined referring to a normal distribution with ±1 σ or ±2 σ. Thus, for example, the costs of hospital treatment were allowed to fluctuate around the mean value of EUR 6600 such that the value used for an alternative model was found to be in the range EUR 5940–7260 with 68.27% probability. For the estimators supported by 95% confidence intervals, these confidence intervals were set equal to ±2 σ, so that values outside the upper/lower limits were also found with decreasing probabilities. To better visualize the influence of individual parameters on the overall result, a one-at-a-time (OAT) sensitivity analysis was performed in addition, in which one parameter of the base model was modified at a time, while all other parameters were kept constant.

## 3. Results

### 3.1. Descriptive Statistics

#### 3.1.1. Development of Case Numbers during the Observation Period

**Figure 3:**
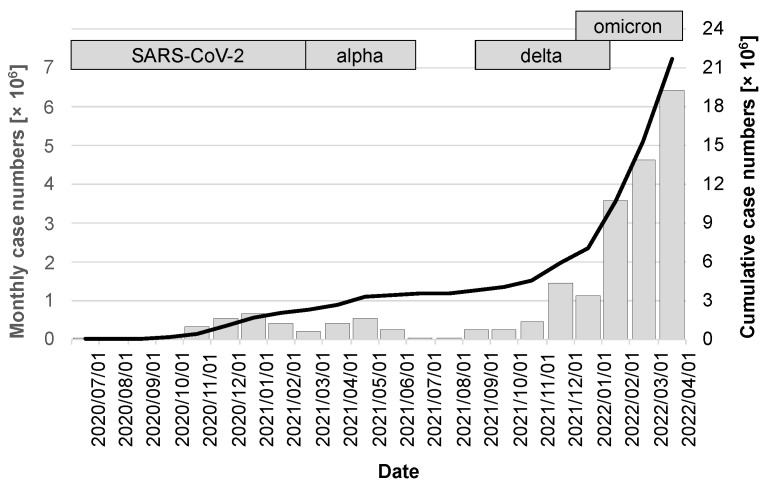
Case numbers in the observation period between release of the CWA and 1 April 2022. The gray bars indicate the cases of the month preceding the day marked on the x-axis and are scaled on the left y-axis. The cumulative case numbers are visualized by the black line with scaling on the right y-axis.

The release of the CWA occurred during a period of very favorable seasonal effects, with low caseloads of just under 17,000 in July 2020, 37,000 in August, and 56,000 in September. A noticeable increase was not seen again until the fall of 2020, peaking at 658,000 cases in December 2020. The first quarter of 2021 then saw a virtually seamless transition to the alpha variant, which, after a moderate drop in the number of cases in February 2021, peaked in April at 546,000 cases. After the summer of 2021 with low case numbers, the delta variant, whose wave also overlapped with the occurrence of the omicron variant, fueled a very dynamic infection activity that led to the highest ever case number in a single month of 6.4 million in March 2022 (gray columns in Figure 3). Over the period under review, there were a cumulative 21.67 million cases (95% CI: 21.66–21.68 million; black line in Figure 3).

#### 3.1.2. Development of the Adoption Rate

In line with the CWA download numbers, the effective adoption rate β_eff_ also increased steadily, reaching its maximum in April 2022 at 24.9% (95% CI: 24.8–25.0%) with a cumulative number of 44.55 million downloads.

#### 3.1.3. Reduction in the R-Value

**Figure 4:**
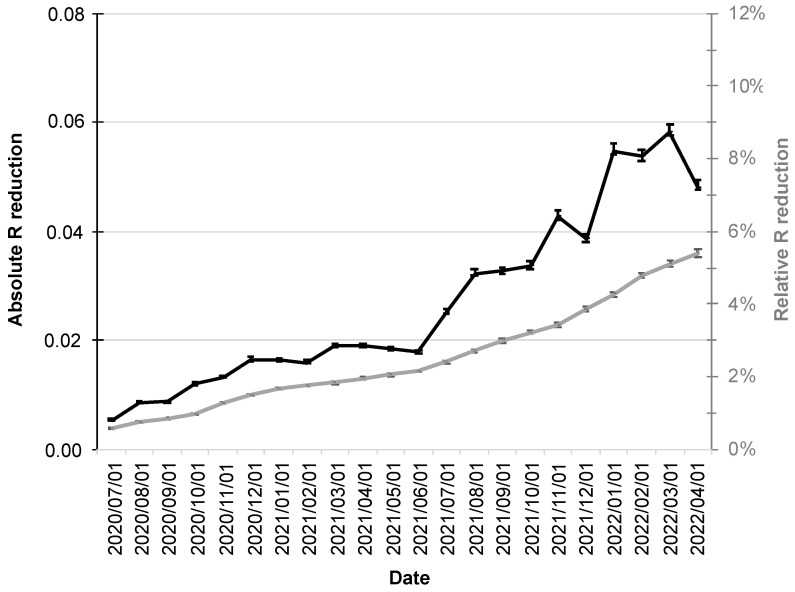
Absolute and relative reduction of the R-value by the CWA. The absolute reduction is shown as a black line scaled on the left y-axis and the relative reduction (%) as a gray line scaled on the right y-axis. The error bars indicate the 95% confidence intervals.

Since increased adoption rates of the CWA are also associated with a stronger influence on the R-value, its relative reduction also increased over the course up to 5.4% (95% CI: 5.3–5.6%; gray line in Figure 4).

The R-value fluctuations over the period under consideration were also reflected in its absolute reductions, which had their minimum at the beginning of the period under consideration (1 July 2020 at 0.0054; 95% CI: 0.0053–0.0056), followed by a moderate increase into December 2020.

The first half of 2021 was then characterized by virtual stagnation with reductions around 0.02. From the second half of 2021, a more pronounced increase in absolute reductions in R-value was followed by a maximum of 0.058 (95% CI: 0.057–0.060; black line in Figure 4) in early March 2022.

### 3.2. Utility Analysis

**Figure 5:**
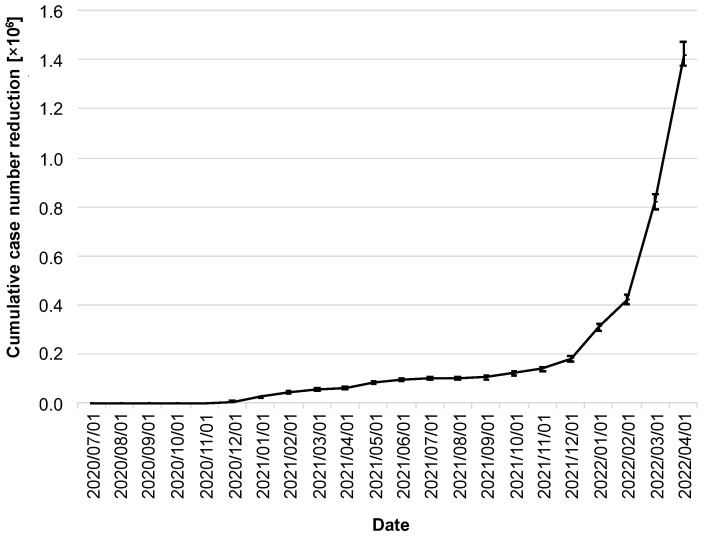
Cumulative reduction of infection cases by the CWA. Data in millions of cases. The error bars indicate the 95% confidence intervals.

The stated goal of the CWA was to help contain the spread of COVID-19. Thus, the most relevant question in this context is to what extent the CWA was able to contribute to the reduction in the number of cases. By 1 April 2022, the CWA had prevented 1.41 million cases (95% CI: 1.38–1.48 million; Figure 5).

It is noticeable that the cumulative reduction in the number of cases stagnated well below 200,000 cases over a long period of time, and, for example, one year after the release of the CWA, there were only 101,000 cases (95% CI: 93,000–107,000; as of 1 July 2021). A massive increase did not begin until the winter of 2021, coinciding with the dynamics of the delta/omicron waves and the steady increase in adoption rates to that point.

The reductions in infection cases due to the CWA were also reflected in reductions in hospitalizations, intensive care treatments, and deaths. These began to manifest themselves in December 2020, reached a plateau in the summer of 2021, and increased dynamically from the winter of 2021.

As of 1 April 2022, the CWA had avoided 17,200 hospitalizations (95% CI: 16,600–18,100), 7200 deaths (95% CI: 6900–7400), and 4600 intensive care treatments (95% CI: 4400–4900; Figure 6).

By this time, the CWA had reduced infection cases by 6.1% (95% CI: 5.8–6.2%), hospitalizations by 4.6% (95% CI: 4.3–4.7%), intensive care treatments by 4.1% (95% CI: 3.8–4.1%), and deaths by 5.6% (95% CI: 5.3–5.7%).

### 3.3. Cost–Benefit Analysis

**Figure 7:**
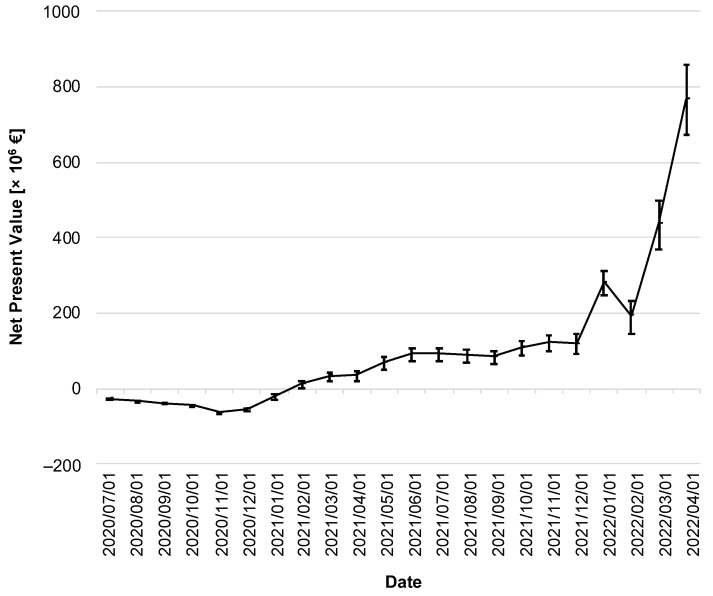
Net present value of the CWA over the reporting period. The discounted net present value is given in million EUR. The error bars indicate the 95% confidence interval.

In the following, a cost–benefit analysis provides information about the CWA’s efficiency. Figure 7 illustrates the change in NPV over time. Immediately after the CWA’s implementation, its costs outweighed the (monetized) benefits, so the NPV of the CWA was negative throughout 2020.

As of February 2021, the lower limit of the 95% CI was in positive territory for the first time (lower limit of the 95% CI: EUR 0.48 million on 1 February 2021), with the NPV subsequently increasing moderately until the fall of 2021. It took until the winter of 2021 to see a sharp increase, ending on 1 April 2022 with an NPV of EUR 765 million (95% CI: EUR 670–860 million).

Among the costs, the CWA-related but negative tests represented the largest single position (EUR 1.44 billion; 95% CI: EUR 1.43–1.45 billion), corresponding to a percentage share of 87.9% of the total costs. On the benefit side, the largest post was due to prevented IfW and the associated reduction in GDP losses (EUR 2.04 billion; 95% CI: EUR 1.95–2.11 billion), corresponding to an 85.0% share of total benefits. 

The BCR of the CWA was determined to be 1.47 (95% CI: 1.41–1.53). Table 2 lists the cumulative discounted costs and benefits over time.

### 3.4. Results of the One-at-a-Time Sensitivity Analysis

**Figure 8:**
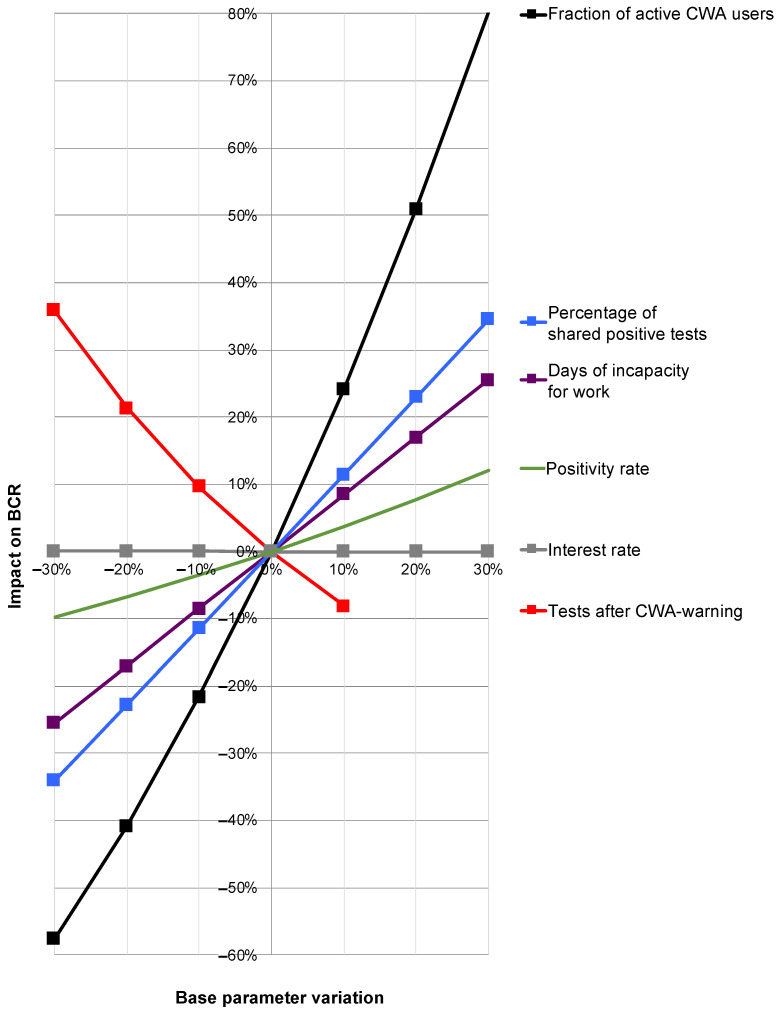
Results of the one-at-a-time sensitivity analysis. Depicted are the percentage variations in the individual base parameters (x-axis) and their percentage impact on the benefit–cost ratio (BCR; y-axis). Red line: rate of perceived testing after red warning by CWA. Purple line: duration of incapacity for work (IfW). Black line: proportion of active users. Blue line: proportion of shared positive test results. Green line: test positivity rate. Gray nearly horizontal line along the x-axis: interest rate.

While the global sensitivity analysis allowed a calculation of the 95% confidence intervals, a change in relevant individual base parameters while keeping all other parameters unchanged provided information about their influence on the overall result by means of an OAT sensitivity analysis (Figure 8). 

Here, a strongly disproportionate positive influence of the share of active users on the BCR was determined. An increase in the proportion of active users by 20% would therefore increase the BCR by around 50% (Figure 8; black line). An increase in the rate of shared positive test results would also have a disproportionate effect on the overall result, albeit to a lesser extent (Figure 8; blue line). There was a slightly under-proportional positive effect for the duration of the IfW (Figure 8; purple line).

For the rate of perceived tests after warning, there was a clear negative correlation with BCR (Figure 8; red line) with a simultaneous at least weak positive correlation of the positivity rate with BCR (Figure 8; green line). Thus, if it were possible to produce fewer red alerts (which turn out to be false alerts) and thereby increase the pretest probability—with a direct effect on the positivity rate—there would be a combined positive effect on the BCR, although this combination cannot be represented in the context of an OAT sensitivity analysis.

The interest rate had no significant effect on the BCR (Figure 8; gray only minimally sloping line almost parallel along the x-axis).

## 4. Discussion

From a health economic perspective, the CWA can be considered a success. As of 1 April 2022, the NPV was clearly in positive territory, and the BCR was well above 1. A positive benefit began to emerge at the beginning of 2021, as rising case numbers and an increasing adoption rate enabled the CWA to unveil its potential. The months prior to this were characterized by barely perceptible benefits—in this period directly after the release, the focus was on the very high costs, even by international standards, which were not yet offset by any significant added value at this time. In retrospect, however, the criticism expressed at this time [49] must be regarded as inaccurate, especially because it was voiced prematurely, even before the positive effects of the CWA could materialize. 

While the CWA then showed a positive NPV from 2021 until the fall of 2021, its full potential was not unleashed until the fall/winter of 2021 with the emergence of the delta and omicron variants. The massive increase in the number of cases in the course of these variants was met by a population increasingly using the CWA, so a large part of the reduction in disease cases fell in these months.

Although the number of intensive care stays and deaths prevented also increased during this phase, it did so to a much lesser extent than the number of cases. This fact is probably due to the milder disease courses caused mainly by the omicron variant compared to the previous virus subtypes [50,51,52,53] and the advanced immunity due to vaccination [54].

From a purely monetary point of view, the greatest potential of the CWA lies in the reduction in the damage to GDP caused by IfW—with a share of 85.0% of the total benefit, this effect makes the benefit of the reduction in direct health care costs such as hospitalizations or intensive care treatments seem almost negligible. On the cost side, the often-criticized position of CWA development and operation is hardly of any importance, accounting for <9% of total costs—the majority of costs are caused by tests that were carried out due to red warnings of the CWA but turned out to be false alarms ex post.

### 4.1. Related Work

While manual contact tracing is considered an established response to infectious disease outbreaks [55,56], DCT apps were first developed in response to COVID-19 and have been studied extensively since then [57]. From the earliest stages, issues of privacy, security, and ethics were raised [58,59,60,61,62,63,64,65], with doubts about privacy identified as the biggest barrier to widespread use of such solutions [60]. Numerous studies investigated potentially promising DCT approaches from technical perspectives, including the currently mainly applied Bluetooth-based method [2,66] but also proximity tracing based on GPS [67], ultrasound [68,69], facial recognition approaches [61], blockchain-based approaches [70], or QR code scanning technology [71].

Apart from these fundamentally different technological approaches, various studies have also been published that examined external influences on the effectiveness of DCT approaches. For example, light-rail trams are known to be potential sites of increased risk of infection, but DCT within the wagons is reduced due to signal reflections caused by the metal structures [72]. Grill et al. [8] examined sociodemographic characteristics of CWA users in Germany and found that app users were less likely to be female, less likely to be younger, and less likely to have lower family incomes but were more likely to live in one of the Western states [8]. From a sociodemographic point of view, there are also studies suggesting a missed communication opportunity regarding the CWA, as many non-users have been found to be unaware of the app’s utility and effectiveness, with the government being criticized for a lack of both transparency and clear communication about its purpose and function [8,73]. A tabular summary of studies on various aspects of DCT apps in general and the CWA in particular can be found in Table 3. As a summarizing comment, although there are studies that attempt to evaluate the effectiveness of DCT applications in general [17], only one study examined caseloads immediately before and immediately after the introduction of various interventions, including DCT apps [74]. There are no studies to date, neither in an international context nor in a national context specifically for Germany, that attempt to estimate the reduction in infection cases and their complications achieved by DCT apps over a longer period of time. There are also no studies that put the costs saved in relation to the costs incurred. Therefore, these are unique aspects of the present study. 

### 4.2. Limitations

One major limitation of the present work is that the introduction of the CWA created a reality that is now being assessed retrospectively. The challenge here lies in modeling the alternative reality and the hypothetical R-value R_hyp_ that would have occurred in the absence of the CWA. This modeling is inherently uncertain, and there are many hidden variables that must be accounted for in such an assessment. Studies on the effectiveness of DCT apps are inconsistent and describe wide ranges of investigated adoption rates, accompanied by correspondingly wide effects on R_eff_ [79]. This fact was taken into account with a regression analysis, the result of which was used for the calculation of R_hyp_. However, the confidence interval of this regression is wide, which is also due to the fact that the individual studies included are heterogeneous and differ with respect to their countries of origin and are essentially based on hypothetical modeling. A transfer of such hypothetical modeling to reality is only possible with certain limitations. In addition, the concept of the adoption rate is handled inconsistently between the individual studies. Moreover, the factor f could only be calculated as a constant but has certainly changed continuously due to varying measures such as lockdowns, relaxations, or immunity as a result of increasing vaccination coverage. For a more precise estimation, however, the available data are, on the one hand, not fine-grained enough and, on the other hand, too complex due to the interaction of various factors (e.g., lockdowns/relaxations, vaccinations, seasonalities, virus variants, public perception of the pandemic situation), so the use of f as a constant represents the best possible, but undoubtedly only limitedly precise, estimation. The inference from R_hyp_ to the hypothetical number of cases (which would have occurred without the CWA) was achieved by means of a linear regression, which, however, is also subject to uncertainties, giving rise to the initially paradoxical fact that a negative reduction in cases was detectable on individual days. The CWA would therefore have led to an increase in the number of cases on these days. However, this phenomenon is purely mathematical—despite the high correlation of the R-value with the rate of new infections, there is the possibility that the inference from R_hyp_ to the hypothetic number of cases and its difference to the real number of cases yields a negative result, just as on other days the number of cases reduction will be overestimated. In total, however, these errors average out in both directions. Another uncertainty arises from the fact that the details of infection chains can only be traced to a limited extent. Due to the pseudonymous tracking of contacts, it is not possible to obtain additional demographic information about users or their activities beyond the mere fact of a contact having taken place, so the analysis cannot be further specified with regard to such criteria either. 

A second limitation of the work is the only limited ability to capture the costs caused by long COVID. Long COVID refers to symptoms that persist between 4 weeks and 6 months after the actual infection has been overcome [80,81]. The fact that hospitalized patients are at higher risk for long COVID symptoms was taken into account by including rehabilitation measures. However, a more detailed estimate of the extent of the long COVID problem beyond this, its evolution especially against the background of ever new virus variants [82,83], and the follow-up costs to be expected in the long-term course over several years remains highly speculative. It therefore seemed sensible to dispense with such speculative estimates and to largely exclude the long COVID problem from the health economic evaluation in the context of this study. However, without being able to even approximate the final long COVID costs, it may be assumed that long COVID cases will continue to generate costs even after the end of this study’s evaluation period, and any avoidance of such cases has therefore further contributed to an increase in the CWA’s NPV.

A third limitation stems from individual details of the accounting and the underlying estimates. Thus, although the evaluation at the cut-off date of 1 April 2022 allowed an objective cost–benefit analysis taking interest rate effects into account, it did not adequately incorporate the reduction in deaths, as these were only considered in terms of the GDP loss avoided until April 2022. Monetizing the value of a life is difficult in any case and gives rise to discussion from an ethical point of view. The literature describes a wide range for the value of a life saved through avoided COVID deaths of USD 1–4.2 million [84,85]. Considering this, even under a very conservative estimate, the more than 7000 deaths prevented by the CWA would have increased the NPV of the CWA by several billion euros—a fact that makes the often-criticized costs of the CWA seem hardly relevant. Moreover, discounting was carried out on a daily basis, which does justice to reality only to a limited extent. For example, the benefit of a prevented hospital treatment was booked on the day of the (prevented) infection, although in reality, costs for hospital treatments are not due on the day of infection, but much later. However, since the interest rate did not have a significant impact on the results in the sensitivity analysis, the effect of inaccurate booking dates can be neglected. With regard to the assessment of indirect costs, it should be noted that in economic evaluations from a societal perspective, this is usually carried out according to the human capital or friction cost approach. Sick pay or pension payments are usually not considered from an economic perspective. In this study, however, average pension payments were nevertheless taken into account, as the valuation of indirect costs of retirees is not handled in a consistent manner in the literature.

Last, the IfW of 22 days per case on average may be overestimated by the fact that a fraction of patients with lighter symptoms may have had shorter periods of lost productivity and the ability to work from home.

Overall, given the required consideration of a large number of variables, it cannot be denied that a certain threat to validity exists within the present analysis. By using publicly available real data, performing a sensitivity analysis including the assumption of realistic variations of all parameters used, and by reporting all results along with their confidence intervals, an attempt was made to reflect and control these uncertainties as best as possible.

### 4.3. Intangible Effects

In addition to monetary effects, the CWA also generates intangible effects, e.g.:

- The reduction in the number of cases of infection caused by the CWA also led to a reduction in suffering—on the one hand, suffering for the patients themselves and, on the other hand, also for their relatives, especially in the case of an otherwise fatal course.
- At least for long COVID cases, but also for other severe courses, the prevention of infection is likely to have contributed to increased quality of life in the longer term.
- The CWA has helped to introduce electronic health solutions on a broad scale and has paved the way for digital health solutions to become part of everyday life.
- The CWA offers a scalable approach, unlike manual contact tracking. German health authorities reach the limits of manual contact tracing at incidences between 35 and 50/100,000 [86]. This fact could only be met to a limited extent and, above all, only approximately linearly by hiring more staff—even by a logistically hardly possible doubling of the staff, only infection cases up to an approximate incidence of 100/100,000 could be followed up manually. DCT apps have no limits even at maximum incidences.
- The concept of the CWA can be used for other pandemics or epidemics in the future with manageable adaptations. There is no doubt that there will be more severe courses of seasonal influenza in the coming years, and another pandemic will almost certainly occur again [87].

These intangible effects add further value to the CWA beyond monetary considerations and round off the view of the CWA’s effectiveness from a more holistic perspective.

### 4.4. Reasons for an Overly Optimistic Assessment

There are reasons why the effectiveness of the CWA may have been incorrectly overestimated in this paper: Preventing infections is highly relevant among the elderly, especially in terms of preventing deaths. However, older people are less tech-savvy due to the phenomenon of technological exclusion. It could therefore be argued that this group of people will be less likely to download or systematically use the CWA [88]; however, studies found an increased uptake among individuals over 50 years of age compared to younger individuals [6]. Another reason for the possibly overly optimistic assessment of the CWA is that both delta and omicron variants of the virus transmit faster than the alpha variant, which is also due to a shortening of the generation time [89,90]. This makes many measures, including contact tracing, testing, and isolation, and thus also the CWA, potentially less effective [10,13]. 

### 4.5. Reasons for an Overly Pessimistic Assessment

However, there are also reasons to believe that the effects of the CWA were erroneously underestimated. The effective adoption rate of the CWA on 1 April 2022 was 24.9%, and the R-value reduction that can be calculated for this adoption rate is 5.4%. In individual studies, however, higher R-value reductions are mentioned for adoption rates up to 40% than would be expected from the regression analysis in this study [10,12,13]. Thus, there is at least the possibility that DCT apps have higher effectiveness at lower adoption rates than assumed in the present work. In addition, the adoption rate in this work was estimated very conservatively by introducing β_eff_. Thus, there is also the possibility that the effectiveness of the CWA could have been higher in reality than calculated in this work.

### 4.6. Possible Optimizations of the Corona Warn App

The fact that a positive test result is shared via the CWA in only 60.1% of cases leads to a notable reduction in its efficiency. If positive test results were instead shared in 95% of cases, the BCR would increase from 1.47 to 2.46, and instead of 1.41 million cases, 2.41 million cases would have been prevented. However, for privacy reasons, emphasis was placed on requiring users to confirm the sharing of a positive test result via the CWA multiple times, which is apparently considered too complicated or causes uncertainty, so this process is often aborted [6,91]. Although the data protection aspect of these considerations is welcome, it can be assumed that sharing positive test results is a central and, above all, indispensable function of the CWA. The informed user could therefore be assumed to be aware of this fact. It would thus be possible, at least in principle, to state in the data protection regulations of the CWA that positive test results are shared automatically. With this measure, the rate of shared positive test results could easily be increased to >95%, with the associated disproportionately positive effects on the effectiveness of the CWA. However, such an approach carries the risk that a larger proportion of users will regard this restriction in data protection as too relevant to continue using the CWA, so such a measure could also have a counterproductive effect.

At the level of the adoption rate, the effectiveness of the CWA could be increased by monetary incentives. In one study, for example, offering even small financial incentives up to a maximum of EUR 5 increased the degree of use by a further 17% [6]. However, such approaches can have a negative impact on intrinsic motivation to use the service, which can also lead to undesirable counterproductive effects. A possible middle ground would be to introduce a kind of lottery among active app users, similar to the approach taken in the U.S. state of Ohio to raise vaccination rates [6,92]. In any case, given the significantly disproportionate impact of app use on its efficiency, raising the number of active users would be highly desirable.

Since the number of tests for infectivity caused by the CWA but negative ex post is a relevant cost factor, other, mainly technical, optimizations of the CWA could aim at minimizing the number of false alarms. For example, it has been shown that a combination of ultrasound technology with the Bluetooth low-energy technology already in use can reduce the number of false positive contacts [69,93]. 

Another positive effect that is difficult to quantify in monetary terms would be the possibility of automatically informing the responsible health authority in the event of a positive test result. In this case, however, only a personalized notification would make sense, which would certainly have to be evaluated more critically from a data protection point of view than the automatic anonymous sharing of a positive test result.

However, potential optimizations of the CWA ultimately represent interventions in an established and currently stable system that may involve uncertain effects on several yet unknown variables. It could therefore be advantageous to keep such changes modest at first and to implement them cautiously under close observation, thus still allowing early and adapted responses to any undesirable effects on the overall system.

## 5. Conclusions

According to the results of the present analysis, the CWA was convincing in terms of both its effectiveness and efficiency. The public perception of the app’s positive effects on pandemic containment is considered the most important factor for its active use [94], which in turn has a strongly disproportionate influence on its benefits—ultimately, this corresponds to a positive feedback loop or self-fulfilling prophecy. 

Given the very high caseloads expected in the fall and winter of 2022, investing in further increasing adoption rates should be considered one of the priority goals for the coming months. This could contribute to another significant reduction in infection cases in the second half of 2022 and early 2023 before the CWA is expected to expire on 31 May 2023 due to ending contracts.

## Figures and Tables

**Figure 6:**
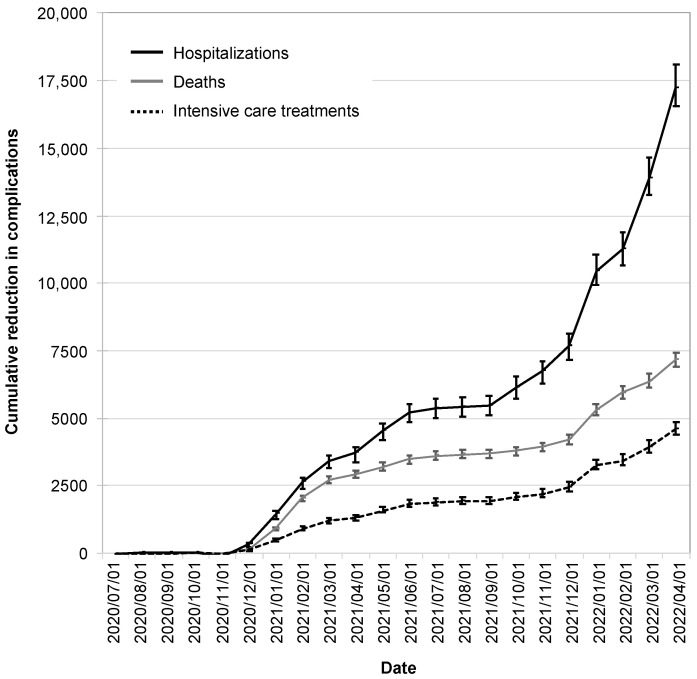
Cumulative reduction in complications by the CWA. Shown are the absolute reductions in hospitalizations (black solid line), deaths (gray line), and intensive care treatments (black dotted line). The error bars indicate the 95% confidence intervals.

**Table 1:** Reduction in R-value as a function of the adoption rates of digital contact tracking apps.

Adoption Rate	Reduction in R_eff_	R_eff_/R_hyp_ in Case of R_hyp_ = 1	Reference
20%	17.6%	0.824	Kretzschmar et al. [10]
40%	20.2%	0.798	
60%	24.4%	0.756	
80%	30.4%	0.696	
100%	38.1%	0.619	
53%	47%	0.53	Kucharski et al. [11]
40%	38.7%	0.613	Plank et al. [12]
60%	40.8%	0.592	
80%	41.7%	0.583	
40%	33.3%	0.667	Elmokashfi et al. [13] ^1^

^1^ In Elmokashfi et al. [13], scenarios with a spectrum of R-values were modeled, with applicable numbers explicitly given for R = 1.5 and R = 2.7. For R = 2.7, however, the authors themselves stated that this extreme scenario was very unrealistic. Since only substantially lower R-values occurred during the period under consideration, only the values for the R = 1.5 scenario were used for the present study.

**Table 2:** Cumulative discounted costs and benefits over time.

Date	Costs, EUR				Benefits, EUR						NPV, EUR
	App	Tests	Pensions	Sum	IfW	HO	IC	Death	Rehab	Sum	
20 July	27	0	0	27	0	0	0	0	0	0	−27
20 August	33	0	0	33	0	0	0	0	0	1	−33
20 September	39	1	0	40	0	0	0	0	0	0	−40
20 October	45	2	0	47	2	0	0	0	0	2	−45
20 November	52	5	0	56	−5	−1	−1	0	−1	−7	−64
20 December	58	12	2	72	10	2	3	1	2	18	−54
21 January	64	20	13	97	40	10	13	5	10	78	−19
21 February	70	27	27	124	68	17	24	10	18	137	13
21 March	76	30	36	141	84	23	32	13	24	175	33
21 April	82	34	38	155	93	25	34	14	26	191	36
21 May	88	44	41	173	124	30	42	15	31	242	69
21 June	94	51	44	188	147	35	48	16	36	281	93
21 July	100	51	45	196	151	36	50	16	37	289	93
21 August	106	53	45	204	153	36	50	16	38	293	89
21 September	112	55	45	213	156	36	50	16	38	297	84
21 October	118	60	46	224	180	41	54	16	42	333	109
21 November	124	75	47	246	204	45	58	17	45	368	123
21 December	130	147	48	325	261	51	64	17	51	444	118
22 January	134	215	51	400	442	69	85	18	69	683	283
22 February	137	474	53	664	600	75	90	19	73	856	192
22 March	141	826	53	1020	1156	92	102	19	88	1457	437
22 April	144	1436	54	1634	2040	114	120	19	106	2399	**765**

Figures in million euros. IfW: Incapacity for work. HO: Hospitalizations. IC: Intensive care. Rehab: Rehabilitation measures. Since the values of the individual columns have been rounded to integers, there may be corresponding discrepancies in the sum and the NPV columns.

**Table 3:** Overview of related work on DCT apps in general and the CWA in particular.

Research Topic	DCT Apps in General	CWA-Specific
Privacy, security, ethics	Afroogh et al. [59] Morley et al. [58] Nabeel et al. [60] Felipe at al. [61] Pratt et al. [62] Nunes et al. [63] Bardus et al. [64]	Afroogh et al. [59] Morley et al. [58] Tomczyk [65]
Technical issues	Ferretti et al. [2] Hatke et al. [66] Shahroz et al. [67] Cranor et al. [68] Loh et al. [69] Felipe at al. [61] Jahmunah et al. [71] Hasan et al. [70]	n/a
Sociodemographic studies	Yeo at al. [75] Chen et al. [76] Dzandu et al. [77]	Grill et al. [8] Amann et al. [73] Horstmann et al. [78] Munzert et al. [6]
Impact of DCT apps on R-value	Jenniskens et al. [17] Kretzschmar et al. [10] Kucharski et al. [11] Plank et al. [12] Elmokashfi et al. [13]	n/a
Impact of DCT apps on absolute caseloads	Leung et al. [74]^1^	Leung et al. [74] ^1^
Cost–Benefit studies	n/a	n/a

^1^ In contrast to the present study, Leung et al. [74] compared caseloads 30 days prior to the release of DCT apps with cases up to 30 days after the release. n/a: no studies available.

## Data Availability

Data supporting the reported results can be found on OSF: DOI 10.17605/OSF.IO/PQMF2.

## Supplementary Materials

The following supporting information can be downloaded at: https://www.mdpi.com/article/10.3390/ijerph192114318/s1, Table S1: Parameters modified in the sensitivity analysis.
