# Supplementary material for: The German COVID-19 Digital Contact Tracing App: A Socioeconomic Evaluation
Source: Int J Environ Res Public Health. 2022 Nov 2;19(21):14318. doi: 10.3390/ijerph192114318 (PMC9654962; doi:10.3390/ijerph192114318)
Supplement: Supplementary file 1 [file ijerph-19-14318-s001.zip › ijerph-1952924-supplementary.pdf]

Table S1. Parameters modified in the sensitivity analysis.

Parameter	Base value	Lower limit	Upper limit
	Financial Mathematics		
Interest rate	0.7%	0.63%	0.77%
	App-related		
Proportion of data donors	58.6%	52.7%	64.5%
Rate of shared positive test results	60.1%	54.1%	66.1%
Rate of performed tests after red warning	87.0%	78.3%	95%
Share of active users in total downloads	59.9%	53.9%	65.9%
Cost of the app	Varying over time, UL/LL modeled with ±10%		
	Disease evolution and demographics		
R-value	Varying over time, UL/LL correspond to 95% CI		
New infections/day	Varying over time, UL/LL correspond to 95% CI		
Proportion of employed persons in infections	Varying over time, UL/LL modeled with ±10%		
Probability per year for a retiree to die independent of COVID-19	4.96%	4.46%	5.5%
Monthly average pension	1,012 €	911 €	1,113 €
Share of retirees in deaths	89.5%	80.6%	95%
Proportion of employed persons in deaths	10.3%	9.3%	11.3%
	R-value modeling		
Constant f used to estimate R-value reduction by the CWA	0.8741	0.3651	1.383
Regression to estimate the infection rate from R_{hyp}			
Slope	0.1064	0.1034	0.1095
y-intercept	-0.04353	-0.04669	-0.04038
	Indirect health care costs		
Isolation period	22 days	14 days	28 days
GDP per capita per year	36,600 €	32,940 €	40,260 €
	Direct health care costs		
Share of patients requiring hospitalization	Varying over time, UL/LL modeled with ±10%		
Share of patients requiring intensive care	Varying over time, UL/LL modeled with ±10%		
Hospitalization costs per patient	6,600 €	5,940 €	7,260 €
Intensive care costs per patient	26,000 €	23,400 €	28,600 €
Daily wage for rehabilitation	211 €	189.90 €	232.10 €
Duration of rehabilitation	27 days	21 days	33 days
Proportion of employed persons requiring rehabilitation	85.2%	83.7%	86.7%
Proportion of intensive care patients in need of rehabilitation	100%	90%	100%
Proportion of hospitalized patients in need of rehabilitation	50%	30%	70%
Cost per PCR test	53.54 €	48.19 €	58.89 €
Cost per rapid test	11.50 €	10.35 €	12.65 €
Test positivity rate	Varying over time, UL/LL modeled with ±10%		

UL/LL: Upper Limit/Lower Limit. CI: Confidence interval.
